# Convenient characterization of sleep EEG in persons at risk or with cognitive decline: realization of a dream?

**DOI:** 10.1093/sleep/zsaf154

**Published:** 2025-06-10

**Authors:** Shuo Qin, Michael W L Chee

**Affiliations:** Centre for Sleep and Cognition, Yong Loo Lin School of Medicine, National University of Singapore, Singapore 117549, Singapore; Centre for Sleep and Cognition, Yong Loo Lin School of Medicine, National University of Singapore, Singapore 117549, Singapore

Sleep tends to deteriorate in older adults and the prevalence of disturbed sleep may be over 50% in persons with neurodegenerative disorders. Individuals with mild cognitive impairment or Alzheimer’s disease (AD) may exhibit prolonged sleep latency, fragmented sleep, reduced slow-wave sleep, and disrupted circadian rhythm [1–3]. Although the associations between sleep problems and impaired cognition are established, there remain open questions regarding the extent to which poor sleep contributes to neurodegeneration, whether it is merely a herald or a marker of decline, or whether interventions to improve sleep can mitigate adverse cognitive outcomes. It is also unknown if treatments for neurodegenerative disorders will improve sleep symptoms. These questions motivate the development of accurate and convenient means of measuring sleep over multiple nights in older adults to determine how established and novel sleep measures relate to disease evolution or its mitigation.

Conventional PSG provides high-quality, brain-relevant data, but is cost- and resource-intensive, making it difficult to upscale, especially for multinight studies in noninstitutional settings. Sleep diaries, questionnaires, and actigraphy have traditionally been used to assess disordered sleep in outpatient settings [4, 5]. However, the former two instruments rely on participant cooperation and recall, while actigraphy has low specificity for wake detection. Single- or limited-channel wireless and wearable EEG devices have emerged to provide sufficiently accurate sleep stage assessment together with EEG power spectra while being easy to deploy in at-home settings [6]. While their features and range of applications re promising [7], high-quality studies documenting the accuracy and clinical usage of these devices remain necessary as their predominant use has been as brain–computer interfaces and for cognitive/fatigue monitoring.

In contrast to its predecessors, the Dreem headband (DHB) records from both occipital and frontal regions, overcoming a key deficiency of frontal-only EEG headbands [8]. It has favorable sleep staging performance (κ ~0.75) when compared to PSG in younger, healthy adults [9, 10]. However, alterations in sleep macro and micro architecture, together with degraded signal quality when studying older adults with cognitive decline, challenge inter-rater agreement of sleep staging for even PSG studies [11].

In this issue, Ravindran et al. [12] present a timely and important comparison of the sleep staging accuracy of DHB compared to PSG in older adults. Participants were either cognitively intact (*n* = 42) or were living with mild AD (*n* = 20). Fundamental sleep metrics: total sleep time (TST), sleep onset latency (SOL), wake after sleep onset (WASO), and sleep efficiency were obtained, together with epoch-by-epoch (EBE) sleep staging and EEG spectral power. There was strong agreement with PSG for TST (ICC = 0.90) and sleep efficiency (ICC = 0.83), with moderate agreement for WASO and SOL. N3 sleep was most accurately detected, while REM and N1 stages were less reliably classified. DHB also showed good correspondence with PSG in estimating delta, theta, and alpha power, particularly during NREM. EBE analysis indicated good sleep-wake discrimination (MCC = 0.77).

Overall close to 75% of recordings were deemed suitable for quantification of sleep spindle and slow-wave metrics. Promisingly DHB performance was not inferior in the group with AD, suggesting it may be used to evaluate cognitively impaired persons. However, a larger sample involving persons with greater sleep disruption would be necessary to substantiate this claim. Additionally, as recordings were conducted in a laboratory under technician supervision, the viability of at-home usage of the DHB remains unclear. For example, users or caregivers putting on these devices may experience difficulties with device pairing or app navigation during unsupervised use [13, 14]. These challenges may affect compliance and data quality essential for evaluating disease course and the effects of intervention. The study also did not assess night-to-night variability in sleep measurements such as sleep efficiency and WASO which can affect executive function and memory in older adults [15–17].

Will the rise in usage of non-EEG wearable and nearable devices affect the utility of wireless EEG headbands? We do not think this is likely in the near term. While such devices should be at least comparable to actigraphy for detecting sleep fragmentation and disruptions in rest–activity patterns [18, 19], the accuracy of their sleep measurement falls off relatively more with the lowered sleep efficiency [10] like that encountered in cognitively impaired persons. Thus, while non-EEG devices can detect subpar sleep, they presently cannot determine the level of impairment at a nightly level. Additionally, EEG-based devices are uniquely positioned to evaluate several less established markers such as NREM hypertonia [20] and microarchitecture features related to spindles or slow oscillations/SWA [6, 21] that are relevant to characterizing sleep in persons with AD.

What additional questions remain before more users embrace dry-electrode wireless EEG headbands? Ravindran’s team evaluated DHB for a single night while the potential of these devices lies in multinight recordings. Such evaluations eliminate the “first-night effect” and provide a long-term record of the cognitive and functional impacts of disrupted sleep and how these might be modulated with treatment. Performance evaluation in at-home settings is sorely needed as with the concurrent evaluation of cognition. Embedding sporadic wireless EEG headband evaluations within long-term studies involving high-quality wearables (or nearables) could help ascertain the discrepancy between non-EEG and EEG-based assessments of sleep macro-architecture for a given individual. Where the discrepancy falls within acceptable limits (what this means remains to be determined), longer-term use of more convenient wearable devices might be supported for that individual.

While the use of DHB for evaluating persons at risk or already with AD is promising, the market for mobile/wearable EEG devices for this purpose is very niche. The relative paucity of studies using these devices compared to the growing number of validation studies on non-EEG wearable sleep trackers indicates a risk of device manufacturers turning their backs on such devices. With this caveat, Ravidran’s article is a valuable addition and one that should stimulate greater interest in using such devices in the study of sleep in older adults at risk of cognitive decline.
